# Supplementary material for: MAFLD in Egyptian non-dialysis CKD Patients: Frequency, fibrosis severity, and risk factors
Source: PLoS One. 2025 Nov 24;20(11):e0336568. doi: 10.1371/journal.pone.0336568 (PMC12643317; doi:10.1371/journal.pone.0336568)
Supplement: S1 Table — (DOCX) [file pone.0336568.s001.docx]

## S1 Table. Results from Firth logistic regression models predicting MAFLD.

| **Variable** | **Coefficient** | **Odds Ratio** | **95% CI** | ***p*-value** |
| --- | --- | --- | --- | --- |
| **Model 1 (DM, HTN; no HbA1c)** | | | | |
| Constant | -0.745 | 0.475 | 0.123 – 1.838 | 0.281 |
| Age (z-score) | -0.246 | 0.782 | 0.411 – 1.486 | 0.452 |
| Sex (Male) | 0.978 | 2.658 | 0.577 – 12.239 | 0.209 |
| **BMI (z-score)** | **1.806** | **6.083** | **2.139 – 17.304** | **<0.001** |
| HOMA-IR (z-score) | 0.902 | 2.465 | 0.568 – 10.702 | 0.229 |
| Diabetes Mellitus (DM) | 1.307 | 3.696 | 0.746 – 18.319 | 0.109 |
| **Hypertension (HTN)** | **1.524** | **4.589** | **1.543 – 13.647** | **0.006** |
| **Model 2 (HbA1c, HTN; no DM)** | | | | |
| Constant | 0.707 | 2.028 | 0.393 – 10.468 | 0.398 |
| Age (z-score) | -0.297 | 0.743 | 0.387 – 1.428 | 0.373 |
| Sex (Male) | 0.342 | 1.408 | 0.290 – 6.828 | 0.671 |
| **BMI (z-score)** | **1.618** | **5.042** | **1.768 – 14.377** | **0.002** |
| HOMA-IR (z-score) | 1.440 | 4.223 | 0.734 – 24.278 | 0.106 |
| Hypertension (HTN) | 0.978 | 2.659 | 0.802 – 8.814 | 0.110 |
| **HbA1c (z-score)** | **1.526** | **4.598** | **1.132 – 18.680** | **0.033** |
| **Model 3 (DM only; no HTN, no HbA1c)** | | | | |
| Constant | 0.036 | 1.036 | 0.304 – 3.529 | 0.954 |
| Age (z-score) | -0.135 | 0.874 | 0.465 – 1.642 | 0.675 |
| Sex (Male) | 1.101 | 3.008 | 0.695 – 13.023 | 0.141 |
| **BMI (z-score)** | **1.844** | **6.320** | **2.296 – 17.395** | **<0.001** |
| HOMA-IR (z-score) | 1.397 | 4.042 | 0.778 – 21.006 | 0.097 |
| Diabetes Mellitus (DM) | 1.569 | 4.804 | 0.954 – 24.194 | 0.057 |
| **Model 4 (HbA1c only; no HTN, no DM)** | | | | |
| Constant | 1.567 | 4.792 | 1.172 – 19.599 | 0.029 |
| Age (z-score) | -0.289 | 0.749 | 0.392 – 1.430 | 0.381 |
| Sex (Male) | 0.152 | 1.164 | 0.249 – 5.452 | 0.847 |
| **BMI (z-score)** | **1.565** | **4.782** | **1.726 – 13.247** | **0.003** |
| **HOMA-IR (z-score)** | **1.968** | **7.157** | **1.180 – 43.419** | **0.032** |
| **HbA1c (z-score)** | **2.001** | **7.396** | **2.014 – 27.163** | **0.003** |

**Notes:**

All models were fit using Firth's bias-reduced logistic regression method. All models were fit on the same cohort of N=108 participants and successfully converged.

**Abbreviations:** CI, Confidence Interval; BMI, Body Mass Index; HOMA-IR, Homeostatic Model Assessment of Insulin Resistance; HbA1c, Glycated Hemoglobin.

Statistically significant results (p < 0.05) are presented in **bold**.
