# Supplementary material for: MAFLD in Egyptian non-dialysis CKD Patients: Frequency, fibrosis severity, and risk factors
Source: PLoS One. 2025 Nov 24;20(11):e0336568. doi: 10.1371/journal.pone.0336568 (PMC12643317; doi:10.1371/journal.pone.0336568)
Supplement: S2 Table — (DOCX) [file pone.0336568.s002.docx]

## **S2 Table. Variance Inflation Factors (VIFs) for Candidate Models.**

Note: All retained models had VIFs <3, indicating no problematic multicollinearity.

| **Model** | **Variable** | **VIF** | **N** |
| --- | --- | --- | --- |
| Model 1 | Age (z) | 1.65 | 108 |
| Model 1 | BMI (z) | 2.01 | 108 |
| Model 1 | Diabetes (DM) | 1.71 | 108 |
| Model 1 | HOMA‑IR (z) | 1.28 | 108 |
| Model 1 | Hypertension (HTN) | 1.22 | 108 |
| Model 1 | Male sex | 1.55 | 108 |
| Model 2 | Age (z) | 1.53 | 108 |
| Model 2 | BMI (z) | 2.15 | 108 |
| Model 2 | HOMA‑IR (z) | 1.28 | 108 |
| Model 2 | HbA1c (z) | 1.70 | 108 |
| Model 2 | Hypertension (HTN) | 1.30 | 108 |
| Model 2 | Male sex | 1.54 | 108 |
