# Supplementary material for: MAFLD in Egyptian non-dialysis CKD Patients: Frequency, fibrosis severity, and risk factors
Source: PLoS One. 2025 Nov 24;20(11):e0336568. doi: 10.1371/journal.pone.0336568 (PMC12643317; doi:10.1371/journal.pone.0336568)
Supplement: S3 Table — (DOCX) [file pone.0336568.s003.docx]

## **S3 Table. Full standard logistic regression outputs (coefficients, ORs, 95% CIs, *p* value, AIC).**

| Model | Variable | Coefficent | OR | CI-lower | CI-upper | *p* value | N | AIC |
| --- | --- | --- | --- | --- | --- | --- | --- | --- |
| Model 1 (DM, HTN; no HbA1c) | Intercept | -0.730 | 0.482 | 0.116 | 2.004 | 0.315 | 108 | 93.70 |
| Model 1 (DM, HTN; no HbA1c) | Age (z) | -0.327 | 0.721 | 0.365 | 1.426 | 0.348 | 108 | 93.70 |
| Model 1 (DM, HTN; no HbA1c) | Male sex | 1.097 | 2.994 | 0.601 | 14.917 | 0.181 | 108 | 93.70 |
| Model 1 (DM, HTN; no HbA1c) | BMI (z) | 2.057 | 7.820 | 2.522 | 24.255 | <0.001 | 108 | 93.70 |
| Model 1 (DM, HTN; no HbA1c) | HOMA‑IR (z) | 1.228 | 3.415 | 0.617 | 18.895 | 0.159 | 108 | 93.70 |
| Model 1 (DM, HTN; no HbA1c) | Diabetes (DM) | 1.652 | 5.217 | 0.869 | 31.331 | 0.071 | 108 | 93.70 |
| Model 1 (DM, HTN; no HbA1c) | Hypertension (HTN) | 1.638 | 5.147 | 1.629 | 16.261 | 0.005 | 108 | 93.70 |
| Model 2 (HbA1c, HTN; no DM) | Intercept | 0.881 | 2.413 | 0.415 | 14.024 | 0.326 | 108 | 91.66 |
| Model 2 (HbA1c, HTN; no DM) | Age (z) | -0.352 | 0.703 | 0.356 | 1.389 | 0.311 | 108 | 91.66 |
| Model 2 (HbA1c, HTN; no DM) | Male sex | 0.373 | 1.453 | 0.274 | 7.693 | 0.661 | 108 | 91.66 |
| Model 2 (HbA1c, HTN; no DM) | BMI (z) | 1.833 | 6.253 | 2.045 | 19.119 | 0.001 | 108 | 91.66 |
| Model 2 (HbA1c, HTN; no DM) | HOMA‑IR (z) | 1.701 | 5.482 | 0.825 | 36.433 | 0.078 | 108 | 91.66 |
| Model 2 (HbA1c, HTN; no DM) | HbA1c (z) | 1.764 | 5.833 | 1.285 | 26.485 | 0.022 | 108 | 91.66 |
| Model 2 (HbA1c, HTN; no DM) | Hypertension (HTN) | 1.057 | 2.877 | 0.820 | 10.094 | 0.099 | 108 | 91.66 |
| Model 3 (DM only; no HTN, no HbA1c) | Intercept | 0.049 | 1.050 | 0.296 | 3.726 | 0.940 | 108 | 99.96 |
| Model 3 (DM only; no HTN, no HbA1c) | Age (z) | -0.180 | 0.835 | 0.434 | 1.610 | 0.591 | 108 | 99.96 |
| Model 3 (DM only; no HTN, no HbA1c) | Male sex | 1.215 | 3.370 | 0.735 | 15.445 | 0.118 | 108 | 99.96 |
| Model 3 (DM only; no HTN, no HbA1c) | BMI (z) | 2.045 | 7.725 | 2.651 | 22.517 | <0.001 | 108 | 99.96 |
| Model 3 (DM only; no HTN, no HbA1c) | HOMA‑IR (z) | 1.561 | 4.765 | 0.827 | 27.450 | 0.081 | 108 | 99.96 |
| Model 3 (DM only; no HTN, no HbA1c) | Diabetes (DM) | 1.881 | 6.562 | 1.116 | 38.572 | 0.037 | 108 | 99.96 |
| Model 4 (HbA1c only; no HTN, no DM) | Intercept | 1.745 | 5.726 | 1.299 | 25.238 | 0.021 | 108 | 92.41 |
| Model 4 (HbA1c only; no HTN, no DM) | Age (z) | -0.328 | 0.720 | 0.371 | 1.400 | 0.334 | 108 | 92.41 |
| Model 4 (HbA1c only; no HTN, no DM) | Male sex | 0.175 | 1.191 | 0.238 | 5.949 | 0.831 | 108 | 92.41 |
| Model 4 (HbA1c only; no HTN, no DM) | BMI (z) | 1.739 | 5.691 | 1.950 | 16.609 | 0.001 | 108 | 92.41 |
| Model 4 (HbA1c only; no HTN, no DM) | HOMA‑IR (z) | 2.163 | 8.700 | 1.311 | 57.741 | 0.025 | 108 | 92.41 |
| Model 4 (HbA1c only; no HTN, no DM) | HbA1c (z) | 2.236 | 9.352 | 2.334 | 37.472 | 0.002 | 108 | 92.41 |
