# Supplementary material for: MAFLD in Egyptian non-dialysis CKD Patients: Frequency, fibrosis severity, and risk factors
Source: PLoS One. 2025 Nov 24;20(11):e0336568. doi: 10.1371/journal.pone.0336568 (PMC12643317; doi:10.1371/journal.pone.0336568)
Supplement: S4 Table — (DOCX) [file pone.0336568.s004.docx]

**S4 Table. Firth bias-reduced logistic regression outputs (coefficients, ORs, 95% CIs, *p* value).**

Notes: Continuous predictors standardized as z-scores. Rows show parameter estimates per model. ‘Converged’ indicates numerical convergence of the penalized likelihood fit.

| Model | Variable | Coefficient | OR | CI-lower | CI-upper | *p* value | N | Converged | Iterations |
| --- | --- | --- | --- | --- | --- | --- | --- | --- | --- |
| Model 1 (DM, HTN; no HbA1c) | Intercept | -0.745 | 0.475 | 0.123 | 1.838 | 0.281 | 108 | Yes | 25 |
| Model 1 (DM, HTN; no HbA1c) | Age (z) | -0.246 | 0.782 | 0.411 | 1.486 | 0.452 | 108 | Yes | 25 |
| Model 1 (DM, HTN; no HbA1c) | Male sex | 0.978 | 2.658 | 0.577 | 12.239 | 0.209 | 108 | Yes | 25 |
| Model 1 (DM, HTN; no HbA1c) | BMI (z) | 1.806 | 6.083 | 2.139 | 17.304 | <0.001 | 108 | Yes | 25 |
| Model 1 (DM, HTN; no HbA1c) | HOMA-IR (z) | 0.902 | 2.465 | 0.568 | 10.702 | 0.229 | 108 | Yes | 25 |
| Model 1 (DM, HTN; no HbA1c) | Diabetes (DM) | 1.307 | 3.696 | 0.746 | 18.319 | 0.109 | 108 | Yes | 25 |
| Model 1 (DM, HTN; no HbA1c) | Hypertension (HTN) | 1.524 | 4.589 | 1.543 | 13.647 | 0.006 | 108 | Yes | 25 |
| Model 2 (HbA1c, HTN; no DM) | Intercept | 0.707 | 2.028 | 0.393 | 10.468 | 0.398 | 108 | Yes | 17 |
| Model 2 (HbA1c, HTN; no DM) | Age (z) | -0.297 | 0.743 | 0.387 | 1.428 | 0.373 | 108 | Yes | 17 |
| Model 2 (HbA1c, HTN; no DM) | Male sex | 0.342 | 1.408 | 0.290 | 6.828 | 0.671 | 108 | Yes | 17 |
| Model 2 (HbA1c, HTN; no DM) | BMI (z) | 1.618 | 5.042 | 1.768 | 14.377 | 0.002 | 108 | Yes | 17 |
| Model 2 (HbA1c, HTN; no DM) | HOMA-IR (z) | 1.440 | 4.223 | 0.734 | 24.278 | 0.106 | 108 | Yes | 17 |
| Model 2 (HbA1c, HTN; no DM) | HbA1c (z) | 1.526 | 4.598 | 1.132 | 18.680 | 0.033 | 108 | Yes | 17 |
| Model 2 (HbA1c, HTN; no DM) | Hypertension (HTN) | 0.978 | 2.659 | 0.802 | 8.814 | 0.110 | 108 | Yes | 17 |
| Model 3 (DM only; no HTN, no HbA1c) | Intercept | 0.036 | 1.036 | 0.304 | 3.529 | 0.954 | 108 | Yes | 15 |
| Model 3 (DM only; no HTN, no HbA1c) | Age (z) | -0.135 | 0.874 | 0.465 | 1.642 | 0.675 | 108 | Yes | 15 |
| Model 3 (DM only; no HTN, no HbA1c) | Male sex | 1.101 | 3.008 | 0.695 | 13.023 | 0.141 | 108 | Yes | 15 |
| Model 3 (DM only; no HTN, no HbA1c) | BMI (z) | 1.844 | 6.320 | 2.296 | 17.395 | <0.001 | 108 | Yes | 15 |
| Model 3 (DM only; no HTN, no HbA1c) | HOMA-IR (z) | 1.397 | 4.042 | 0.778 | 21.006 | 0.097 | 108 | Yes | 15 |
| Model 3 (DM only; no HTN, no HbA1c) | Diabetes (DM) | 1.569 | 4.804 | 0.954 | 24.194 | 0.057 | 108 | Yes | 15 |
| Model 4 (HbA1c only; no HTN, no DM) | Intercept | 1.567 | 4.792 | 1.172 | 19.599 | 0.029 | 108 | Yes | 12 |
| Model 4 (HbA1c only; no HTN, no DM) | Age (z) | -0.289 | 0.749 | 0.392 | 1.430 | 0.381 | 108 | Yes | 12 |
| Model 4 (HbA1c only; no HTN, no DM) | Male sex | 0.152 | 1.164 | 0.249 | 5.452 | 0.847 | 108 | Yes | 12 |
| Model 4 (HbA1c only; no HTN, no DM) | BMI (z) | 1.565 | 4.782 | 1.726 | 13.247 | 0.003 | 108 | Yes | 12 |
| Model 4 (HbA1c only; no HTN, no DM) | HOMA-IR (z) | 1.968 | 7.157 | 1.180 | 43.419 | 0.032 | 108 | Yes | 12 |
| Model 4 (HbA1c only; no HTN, no DM) | HbA1c (z) | 2.001 | 7.396 | 2.014 | 27.163 | 0.003 | 108 | Yes | 12 |
