# Supplementary material for: MAFLD in Egyptian non-dialysis CKD Patients: Frequency, fibrosis severity, and risk factors
Source: PLoS One. 2025 Nov 24;20(11):e0336568. doi: 10.1371/journal.pone.0336568 (PMC12643317; doi:10.1371/journal.pone.0336568)
Supplement: Attachment — Submitted filename: Review of Article-20250910.docx [file pone.0336568.s008.docx]

**Summary:**

This cross-sectional study investigated the prevalence and risk factors of metabolic dysfunction-associated fatty liver disease (MAFLD) among 108 Egyptian non-dialysis chronic kidney disease (CKD) patients. MAFLD was present in 59.25% of patients. Compared with non-MAFLD patients, those with MAFLD were older, had higher BMI, and more frequently had diabetes, hypertension, and insulin resistance. They also showed lower eGFR and greater liver stiffness, with advanced fibrosis more common in later CKD stages. These findings highlight the strong metabolic and renal risk overlap, underscoring a bidirectional relationship between MAFLD and CKD. Early screening and integrated management strategies are essential to mitigate disease progression and improve patient outcomes in this high-risk population. However, there are still lots of comments which need to be addressed by the author.

**Comments:**

1. Terminology (MAFLD vs MASLD/MASH): Since 2023 many societies adopted MASLD (and MASH for steatohepatitis). You use MAFLD and MASH—this mixes framework. Either (a) justify sticking with MAFLD and avoid MASLD/MASH terms, or (b) adopt MASLD/MASH consistently and cite the consensus.
2. The author motivated the study by Egypt’s high metabolic burden but provide no local data (obesity, T2D, CKD prevalence, prior MAFLD estimates in Egypt). Include 1–2 Egypt-specific stats to justify the population focus.
3. The author mentioned that findings are “conflicting” but don’t specify what conflicts (incidence vs severity association? effect after adjusting for obesity/T2D?). Add 1–2 concrete examples to make the knowledge gap explicit.
4. “≥5% fat accumulation” is vague in an ultrasound context. Consider “≥5% hepatic steatosis by histology or its noninvasive surrogates (e.g., CAP/MRI-PDFF)”—or defer the operationalization to Methods and keep the intro conceptual.
5. Line 106, replace “has emerged as the most prevalent” with “is the most prevalent” (if cited), or “is among the most prevalent” if uncertain.
6. Alcohol exclusion conflicts with MAFLD. MAFLD does not exclude alcohol by definition (that was NAFLD). Excluding “any alcohol consumption” can bias the sample; either justify or align with MAFLD.
7. Viral hepatitis is not explicitly excluded/tested. In Egypt this is essential. Specify HBsAg/anti-HCV testing (and exclusion if positive).
8. Metabolic criteria mix ethnic cutoffs that don’t fit Egyptians. You use “white vs Asian” thresholds for BMI and waist. Provide Middle East/North Africa–appropriate cutoffs (or justify using Europid 94/80 cm; BMI ≥25).
9. Clarify whether G5 non-dialysis patients were included; if yes, describe how you handled potential confounding from uremia on labs and elastography feasibility.
