# Supplementary material for: MAFLD in Egyptian non-dialysis CKD Patients: Frequency, fibrosis severity, and risk factors
Source: PLoS One. 2025 Nov 24;20(11):e0336568. doi: 10.1371/journal.pone.0336568 (PMC12643317; doi:10.1371/journal.pone.0336568)
Supplement: Attachment — Submitted filename: Response to Reviewer Comments.docx [file pone.0336568.s009.docx]

**Response to Reviewer Comments**

**Manuscript ID:** PONE-D-25-30973

**Title:** *MAFLD in Egyptian Non-Dialysis CKD Patients: Frequency, Fibrosis Severity, and Risk Factors*

Dear Editor and Reviewer,

We would like to thank the reviewer for their careful reading of our manuscript and their constructive comments, which have helped us to improve the clarity, accuracy, and rigor of the paper. Below, we provide a detailed point-by-point response. Reviewer comments are in **bold**, and our responses follow.

**1. Terminology (MAFLD vs. MASLD/MASH)**

**Comment:** Since 2023 many societies adopted MASLD (and MASH for steatohepatitis). You use MAFLD and MASH—this mixes frameworks. Either (a) justify sticking with MAFLD and avoid MASLD/MASH terms, or (b) adopt MASLD/MASH consistently and cite the consensus.

**Response:** We thank the reviewer for raising this important point. In our study we deliberately used the **MAFLD** terminology, which remains widely cited in clinical research, especially in nephrology-focused literature. To avoid confusion, we have:

- Revised the manuscript to consistently use *MAFLD* only (omit MASH in the introduction).
- Added clarification in the Introduction noting that although MASLD has been recently proposed, MAFLD is still prevalent in research contexts and reflects the criteria we applied in patient recruitment.
- Included the relevant consensus reference (Eslam et al., 2023).

**2. Local data to justify Egyptian focus**

**Comment:** The author motivated the study by Egypt’s high metabolic burden but provide no local data (obesity, T2D, CKD prevalence, prior MAFLD estimates in Egypt).

**Response:** We agree this contextualization is important. We have now added Egypt-specific statistics:

- National obesity prevalence (36% adults, WHO 2022).
- T2D prevalence (~17%, IDF 2021).
- CKD prevalence estimates (~15% among adults, Gawad et al., 2024).
  These are cited in the Introduction to strengthen the rationale for studying Egyptian CKD patients.

**3. Conflicting findings not specified**

**Comment:** The author mentioned that findings are “conflicting” but don’t specify what conflicts.

**Response:** We clarified in the Introduction that prior studies have reported:

- Some showing MAFLD independently increases CKD incidence and fibrosis risk (e.g., Musso et al., 2014).
- Others finding no independent effect after adjusting for obesity and diabetes (e.g., Mantovani et al., 2018).

This revision explicitly identifies the knowledge gap our study addresses.

**4. Definition of ≥5% fat accumulation**

**Comment:** “≥5% fat accumulation” is vague in ultrasound context.

**Response:** We revised the Introduction to state: *“Hepatic steatosis defined histologically as ≥5% fat accumulation, or by non-invasive surrogates such as ultrasound, CAP, or MRI-PDFF in clinical practice”*. Operational definitions remain in the Methods.

**5. Intro wording (line 106)**

**Comment:** Replace “has emerged as the most prevalent” with “is the most prevalent.”

**Response:** Corrected as suggested.

**6. Alcohol exclusion vs. MAFLD definition**

**Comment:** Alcohol exclusion conflicts with MAFLD definition.

**Response:** We thank the reviewer for this observation. In our setting, alcohol consumption is rare (<1%) due to sociocultural factors. For methodological clarity, we excluded patients with *any alcohol intake* to avoid diagnostic ambiguity. We clarified this rationale in the Methods.

**7. Viral hepatitis exclusion**

**Comment:** Viral hepatitis not explicitly excluded/tested.

**Response:** We have now clarified in the Methods that all participants underwent **HBsAg and anti-HCV testing**, and those positive were excluded. This ensures the cohort represents purely metabolic liver disease.

**8. Ethnic cutoffs for BMI/Waist circumference**

**Comment:** The manuscript uses Caucasian/Asian cutoffs.

**Response:** We have corrected this by adopting **Middle East/North Africa–appropriate cutoffs** (BMI ≥25 kg/m² for overweight, ≥30 kg/m² for obesity; waist ≥94 cm men, ≥80 cm women, per IDF). These are now clearly stated and referenced.

**9. CKD stage G5 inclusion**

**Comment:** Clarify whether non-dialysis G5 patients were included.

**Response:** Yes, non-dialysis G5 patients were included (n=10). We have clarified this in the Methods. We also acknowledge in the Discussion that uremia may affect laboratory and elastography results, which we treated as a limitation.

**Closing Statement**

We have revised the manuscript to address all comments, and we believe these changes strengthen its clinical and methodological clarity. We are grateful for the reviewer’s insightful feedback.

Sincerely,
**Hesham Kamal Habeeb Keryakos, MD, PhD**

On behalf of all co-authors
