# Supplementary material for: MAFLD in Egyptian non-dialysis CKD Patients: Frequency, fibrosis severity, and risk factors
Source: PLoS One. 2025 Nov 24;20(11):e0336568. doi: 10.1371/journal.pone.0336568 (PMC12643317; doi:10.1371/journal.pone.0336568)
Supplement: Attachment — Submitted filename: Response to Reviewer Comments.pdf [file pone.0336568.s010.pdf]

Response to Reviewer Comments

Manuscript ID: PONE-D-25-30973R1
Title: MAFLD in Egyptian Non-Dialysis CKD Patients: Frequency, Fibrosis Severity, and Risk Factors

Dear Editor and Reviewers,

We sincerely thank the reviewers for their careful assessment and thoughtful comments, which substantially improved the clarity and rigor of our manuscript. Below, we provide a point-by-point response and describe the corresponding revisions made in the manuscript.

Reviewer #1

Overall comment: "Well-structured with appropriate methodology, but requires further revisions prior to publication."

1) "Paradoxical" protective effects for hypertension (HTN) and diabetes (DM) suggest model misspecification

Comment: HTN (OR≈0.13) and DM (OR≈0.14) showing protective associations is suspicious.

Response: We agree these estimates warranted a re-specification of our models to reduce redundancy and potential suppression effects. We therefore:

- Refit the logistic regression using alternative, non-redundant covariate sets to avoid overlapping constructs (e.g., modeling with either DM or HbA1c, but not both simultaneously; similarly, modeling HTN separately from RAAS-inhibitor use).
- Centered and standardized continuous predictors (BMI, HbA1c, HOMA-IR, age) to improve numerical stability.
- Performed a **Firth penalized logistic regression** sensitivity analysis to guard against small-sample bias in some strata.

In the **revised Table 5**, HTN and DM no longer display paradoxical "protective" directions; estimates and 95% CIs now align with clinical plausibility. We reference this change in the Results and note the re-specification details in the Methods (Statistical Analysis). (See revised regression section and tables in the manuscript.)

2) Possible multicollinearity among BMI, DM, HTN, HOMA-IR, HbA1c

Response: We computed **Variance Inflation Factors (VIFs)** for all candidate models and report them in **Supplementary Table S2**. Across the final models, VIFs were below commonly accepted thresholds (all <3), indicating no problematic multicollinearity after re-specification. We also added a brief paragraph in Methods (Statistical Analysis) describing multicollinearity checks and model selection criteria. (See updated Methods and Supplement.)

3) Medication-adjusted ORs (Table 6) appear post-hoc; methodology unclear

Response: We appreciate this and have fully clarified the approach. **Table 6 now presents results from a distinct multivariable model ("Model 2")** that *simultaneously includes* medication variables (RAAS inhibitors, statins, insulin therapy, diuretics) alongside the core covariates retained after re-specification. We removed any language implying post-hoc "estimate adjustments."

- Methods now specify: model formulas, covariate entry criteria, and how medication use was encoded.
- **Medication-inclusive model.** To examine medication effects without introducing overlapping constructs, we specified a distinct **Model 2 (medication-inclusive)** that adds **RAAS inhibitor use, statins, insulin therapy, and diuretics** to the re-specified core covariate set. Because insulin therapy directly relates to glycemic control, we **modeled glycemia with standardized HbA1c (z-score) rather than a binary DM indicator** in this specification. The final Model 2 covariates were: **age (z), sex (male=1), BMI (z), HOMA-IR (z), HbA1c (z), HTN (yes=1), RAAS inhibitors (yes=1), statins (yes=1), insulin therapy (yes=1), diuretics (yes=1).**
- **Model formula.** For participant i, the medication-inclusive model was:

$$\begin{aligned}\operatorname{logit} P(\mathrm{MAFLD}_i = 1) \\ &= \beta_0 + \beta_1 \mathrm{Age}_z + \beta_2 \mathrm{Male} + \beta_3 \mathrm{BMI}_z + \beta_4 \mathrm{HOMA\text{-}IR}_z + \beta_5 \mathrm{HbA1c}_z + \beta_6 \mathrm{HTN} \\ &+ \beta_7 \mathrm{RAASi} + \beta_8 \mathrm{Statin} + \beta_9 \mathrm{Insulin} + \beta_{10} \mathrm{Diuretic}.\end{aligned}$$

- We harmonized labeling in Table 6 (no “Estimated OR” phrasing) and cross-referenced both models in the Results.

4) Early-stage CKD subgroup (n=16) underpowered

Response: We agree. We now explicitly acknowledge that **early-stage CKD comparisons are underpowered** and treat those analyses as exploratory. This limitation is highlighted in the Discussion and Limitations, and we’ve tempered the language of inference accordingly. (See revised Discussion and figure captions.)

5) Make limitations more explicit

Response: We expanded the **Limitations** section to emphasize: (i) cross-sectional design (no causal inference), (ii) single-center setting, (iii) reliance on non-invasive fibrosis tools (with CKD-related constraints), (iv) potential residual confounding, and (v) limited power in certain strata. (See revised Discussion.)

6) Typos/wording fixes

Response: Corrected as suggested.

- Line 414: “wose liver fibrosis” → “worse liver fibrosis.”
- Line 500: “relevent” → “relevant.”
- We also performed a thorough proofread for consistency and grammar.

7) CKD prevalence reference (Gawad et al., 2024) may not represent national prevalence

Response: We revised the text to **avoid implying national representativeness** for this single-center report and retained it only as **contextual local data**. To frame the burden more appropriately, we now pair local figures with **global/consensus CKD epidemiology** (e.g., Kovesdy 2022; KDIGO/KDOQI commentary) and **WHO/IDF** sources for obesity/T2D, clarifying scope and uncertainty. (See updated Introduction and references.)

Reviewer #2

Comment: “Thorough revision; suitable for publication.”

Response: We thank the reviewer for the positive assessment. We maintained the clarified methods/statistics, improved tables/figures list, and tempered conclusions as in R1, and further addressed Reviewer #1's statistical concerns in the present revision. (See Lists of Tables/Figures and statistical sections.)

Additional editorial compliance notes

- **Terminology (MAFLD vs MASLD/MASH):** We consistently use **MAFLD** throughout and explain the rationale and timing relative to recruitment, citing consensus and guidance. (See Introduction and the prior R1 response block.)
- **Exclusions (alcohol, viral hepatitis) and ethnic cut-offs:** These are now clearly stated in Methods (with Middle East/North Africa–appropriate BMI/waist thresholds, and HBsAg/anti-HCV testing) as already reflected in R1.
- **Data availability:** The R1 PDF includes a **Zenodo DOI** for the dataset, ensuring full compliance with PLOS ONE data policy (the DOI appears in the manuscript). We reiterate this in the Data Availability statement.

Summary of key manuscript changes (tracked in the revised file)

1. **Statistical Analysis (Methods):** Added model re-specification details, VIF assessment, Firth sensitivity, and explicit description of "Model 2" including medications.
2. **Results:** Updated **Table 5** (primary multivariable model) and **Table 6** (medication-inclusive model) with full clarity; paradoxical directions resolved; language tempered for exploratory subgroup results.
3. **Supplement:** Added **Table S2 (VIFs)** and **Table S1 (full regression outputs)**.
4. **Discussion/Limitations:** Expanded limitations (design, single-center, non-invasive fibrosis assessment in CKD, residual confounding, small strata).
5. **Proofing:** Corrected typographical errors and tightened language as flagged.

We are grateful for the reviewers' guidance. We believe these revisions substantially enhance the robustness and transparency of our work.

Sincerely,
Hesham Kamal Habeeb Keryakos, MD, PhD (Corresponding Author)

On behalf of all co-authors
